# The quality of preventive health care delivered to adults: results from a cross-sectional study in Southern Italy

**DOI:** 10.1186/1471-2458-10-350

**Published:** 2010-06-18

**Authors:** Benedetto Manuti, Paolo Rizza, Aida Bianco, Carmelo GA Nobile, Maria Pavia

**Affiliations:** 1 Chair of Hygiene, Medical School, University of Catanzaro "Magna Græcia", Catanzaro, Italy

## Abstract

**Background:**

It is assumed that providing clinical preventive services to patients can identify or detect early important causes of adult mortality. The aim of this study was to quantify access to preventive services in Southern Italy and to assess whether and how the provision of preventive care was influenced by any specific characteristics of patients.

**Methods:**

In a cross-sectional study adults aged 18 years and over attending primary care physician (PCP) offices located in Southern Italy were interviewed from June through December 2007. Quality indicators of preventive health care developed from RAND's Quality Assessment Tools and Behavioral Risk Factor Surveillance System (BRFSS) were used. Multivariate analysis was performed to identify and to assess the role of patients' characteristics on delivery of clinical preventive services.

**Results:**

A total of 1467 subjects participated in the study. Excepting blood pressure preventive check (delivered to 64.4% of eligible subjects) and influenza vaccination (recommended to 90.2% of elderly), the rates of delivery of clinical preventive services were low across all measures, particularly for screening and counseling on health habits. Rates for providing cancer screening tests at recommended times were 21.3% for colonoscopy, 51.5% for mammography and 52.4% for Pap smear. Statistical analysis showed clear disparities in the provision of clinical preventive services associated with age, gender, education level, perceived health status, current health conditions and primary care access measures.

**Conclusions:**

There is overwhelming need to develop and implement effective interventions to improve delivery of routine clinical preventive services.

## Background

Scientific consensus holds that the provision of clinical preventive services to adults can identify and detect many causes of adult mortality. Despite the fact that prevention is a core component of primary care practice, there is widespread evidence that clinical preventive service delivery rates are low and generally sub-optimal [1].

To assess the extent to which the healthcare system succeeds in providing essential clinical preventive services, composite measures have been developed. Among these measures, the RAND's Quality Assessment Tools system has been extensively used to assess the quality of certain preventive services which are routinely recommended for adults regarding screening and counseling on health habits, physical examination, vaccinations and screening for cancers [2]. Previous studies in USA have found significant disparities in the provision of these clinical preventive services, and these disparities are associated with the patients' characteristics, such as sex, age, race or ethnic group, income and health insurance [3-6].

In Italy, where universal and free access to interventions included in the Basic Healthcare Parameters is provided through the National Health Service (NHS), financial resources for prevention are very limited; furthermore, preventive healthcare that is provided by Prevention Departments and primary care physicians (PCPs), is often not properly organized. Moreover, during the 1990s the Italian NHS was decentralized into twenty independent regions, thus increasing the risk of inequality in access to health in the more impoverished places of the South. Indeed, studies have demonstrated a striking imbalance in the provision of preventive health services, especially in breast, cervical and colorectal cancer screening, with higher coverage always in Northern and Central Italy compared to Southern Italy, where organised systematic screening activity has not yet been completely implemented [7-9].

Several studies have been published which focused on a narrow set of quality indicators and conditions in selected populations, but they showed limited ability to evaluate the role of specific factors associated with poorer quality [10-12]. Very few studies have examined the quality of preventive care for multiple conditions [13-16]. In the present study, we intend to quantify the access to various preventive services in Southern Italy, and to identify the relationships between patients' characteristics and the quality of preventive care which is supplied by PCPs.

## Methods

### Study population

A cross-sectional study was conducted at waiting rooms of 20 PCPs in two cities of Southern Italy (Catanzaro and Crotone), randomly selected from lists provided by the Local Health Units. Catanzaro had 60 PCPs and Crotone 38. These two cities, Catanzaro and Crotone, are located in the Calabria Region (2 million inhabitants as a whole) in the extreme southeastern part of Italy; the former is the capital of the region and the latter is a small town, yet they have common urban and demographic characteristics typical of the southern region of the country. The adult population was 77,022 in Catanzaro and 44,047 in Crotone. From June through December 2007 two trained physicians gathered information from adult patients attending PCPs who consented to participate, to determine "technical process quality" (whether or not patients were offered recommended services for which they were eligible) across a wide spectrum of preventive healthcare services. On average, 30 patients were attending consultations on each occasion. All information was self-reported by the participants. No medical records or interviews by any PCPs were used as sources of data.

Sample size calculation assumed the worst scenario of an estimated proportion of each characteristic of interest in 50% of the population. As we were interested in at least 200 subjects in each subgroup within the sample for each separate estimate required, we decided to be conservative and to increase our sample size to 1500 subjects so that we would have sufficient precision in the estimates on the whole sample.

### Review instrument

The RAND Quality Assessment Tools system is a composite of 35 quality indicators, of which we selected 15 [2]: to assess potential problems with underuse of the following preventive services routinely recommended for adults: screening and counseling on health habits (sexual activity, drug abuse, alcohol consumption and smoking cessation), physical examination (blood pressure check and evaluation of hearing difficulties), vaccinations against influenza and pneumococcal disease (in all patients 65 yrs and over and in patients younger than 65 yrs with conditions that represent a risk for severe complications in subjects undergoing influenza and pneumococcal disease) and recommended screening for early diagnosis of cancers according to age and gender (breast, cervical and colorectal). (See Additional file 1). The decision to exclude 20 indicators was suggested for several reasons:

1) we excluded 11 indicators because they needed to be verified by medical records; 2) we excluded 8 indicators that pertained to subgroups of populations that would be rarely encountered in our setting and would therefore offer low numbers of eligible subjects (for example, pregnant women not immune to rubella or women with a history of cervical dysplasia or carcinoma-in-situ or HIV infection who have not had Pap smear within the past year ); 3) we excluded one indicator that predictably would have an absolute 100% failure rate (that is, counseling regarding the use of seat belts).

We defined chronic diseases (using WHO criteria) as diseases of long duration and generally slow progression, such as heart disease, stroke, cancer, chronic respiratory diseases, diabetes etc. [17], and we classified participants as having chronic diseases if they reported ever being advised by a healthcare worker that they had a chronic disease and/or if they were currently in treatment for these conditions. Similarly, we defined participants as hypertensive if they reported ever being advised by a healthcare worker that they had high blood pressure and/or if they were taking an antihypertensive medication.

Additional questions were derived from the BRFSS 2007 Questionnaire [18]. These evaluated smoking and alcohol consumption habits, as well as recommendations of physicians on how to reduce behavioral risk factors related to high blood pressure, and on compliance of patients to colorectal, breast and cervix cancer screening. We defined current smokers as people who reported smoking at least 100 cigarettes during their lifetime and who currently smoke every day or some days. Current drinking was defined as consuming alcohol on one or more of the previous 30 days. Heavy drinking was defined as an average consumption of more than 2 drinks per day during the previous 30 days among men and more than 1 drink per day during the previous 30 days among women (>30 drinks/month). Excessive alcohol use was defined on the basis of either heavy drinking (high average consumption), binge drinking (high per occasion consumption), or both [19].

The interview was also intended to collect the following data for each patient: socio-demographics (age, sex, marital status, number of persons in the household, education level, working activity), perception of health status and utilization of health services during the previous year (specialist visits, emergency accesses, and hospital admissions).

We used level of education as a proxy indicator for socioeconomic status (SES) for several reasons, such as ease of measurement, applicability to persons not in the active labor force (e.g. homemakers, unemployed, and retired), and stability over adult lifespan. We preferred this definition instead of measuring income, since subjects consider this information too sensitive, and as a result they may offer less truthful responses. The perception of health status was assessed by using two components, *12-Physical Component Summary (PCS-12) *and *12-Mental Component Summary (MCS-12*), from the Medical Outcomes Study's 12-Item Short-Form Survey Instrument, *SF-12*, in its validated Italian version [20-22].

The study protocol was approved by Ethics Committee of the "Mater Domini" Hospital of Catanzaro (Italy) (Prot. E.C. n. 127/2006).

### Statistical analysis

Multivariate stepwise logistic regression analysis was performed. Six models were developed including those variables potentially associated with the following outcomes of interest: blood pressure check on all patients otherwise presenting for care at least once each year (Model 1) (0 = no, 1 = yes); colorectal cancer screening with sigmoidoscopy or colonoscopy within the previous 10 years received by subjects aged 50 to 80 (Model 2) (0 = no, 1 = yes); cervical cancer screening received within the previous 3 years by all sexually active women (Model 3) (0 = no, 1 = yes); breast cancer screening received in the previous 2 years by women aged 50 to 70 (Model 4) (0 = no, 1 = yes); influenza and pneumococcal vaccinations offered at recommended times to subjects aged 65 and over (Model 5) (0 = no, 1 = yes); alcohol problem screening received by all subjects (Model 6) (0 = no, 1 = yes). In all models the explanatory variables included were the following: gender (male = 0, female = 1) when appropriate, age (continuous), marital status (married = 0, other = 1), additional persons in the household (ordinal: none = 0, 1 = 1, >1 = 2), education level (ordinal: no formal education = 1, primary school = 2, secondary school = 3, high school or higher = 4), working activity (housewife/student/unemployed/retired = 1, other = 2), chronic disease (none = 0, ≥1 = 1), PCS score (continuous), MCS score (continuous), current tobacco use (ordinal: not at all = 0, some days = 1, every day = 2), excessive alcohol use (no = 0, yes = 1), PCP accesses in the previous year (<1 time per month = 0, ≥1 time per month = 1), PCP medical visits in the previous year (ordinal: none = 0, <1 time per month = 1, ≥1 time per month = 2), specialist visits in community health services (none = 0, ≥1 = 1), private specialist visits (none = 0, ≥1 = 1), emergency accesses in the previous year (none = 0, ≥1 = 1) and hospital admissions in the previous year (none = 0, ≥1 = 1). The significance level for variables entering the logistic regression models was set at 0.2 and for removal from the model at 0.4. Adjusted odds ratio (ORs) and 95% confidence intervals (CIs) were calculated. The data were analyzed using the Stata software program, version 10.1 (Stata Corporation. College Station, TX) [23].

## Results

Of 1716 adults who were eligible for the study, 1467 agreed to participate and were enrolled, for a response rate of 85.5%. The main characteristics of the study population regarding socio-demographic profile, health condition and utilization of health services are presented in Table 1. Of all participants, 50.7% were current or ex-smokers; current drinkers (with an average of 1.5 drinks) and excessive drinkers were 58.1% and 8.3%, respectively. The mean score of perceived health status was 45.9 for PCS-12 and 44.9 for MCS-12. Of the participants, 46.7% had access at least monthly to a PCP, 39.3% and 47% had at least one specialist visit to the public or private sector, respectively; 23% and 12.8% reported at least one emergency visit and one hospital admission, respectively.

**Table 1:** Selected characteristics of the study population

Characteristic	N (%)	Mean (±SD)
**Sex**		
Male	660 (45)	
Female	807 (55)	
**Age group, years**		52.3 (±17.0)
18-45	498 (33.9)	
46-64	541 (36.9)	
≥ 65	428 (29.2)	
**Education level**		
No formal education	305 (20.8)	
Primary school	309 (21)	
Secondary school	588 (40.1)	
University degree	265 (18.1)	
**Marital status**		
Married	988 (67.3)	
Other	479 (32.7)	
**Living condition**		
With family	1020 (69.5)	
Other	447 (30.5)	
**Additional persons in the household**		
None	138 (9.4)	
1	384 (26.2)	
>1	945 (64.4)	
**Working activity**		
No	843 (57.5)	
Yes	624 (42.5)	
**Chronic diseases**		
No	579 (39.5)	
Yes	888 (60.5)	
**Hypertension**		
No	972 (66.3)	
Yes	495 (33.7)	
**Tobacco use in entire life**		
No	724 (49.3)	
Yes	743 (50.7)	
**Frequency of current smoking**		
Not at all	360 (48.4)	
Some day	37 (5)	
Every day	346 (46.6)	
**Attempts to quit smoking in entire life among current smokers**		
No	184 (48)	
Yes	199 (52)	
**Attempts to quit smoking in the previous year among current smokers**		
No	109 (54.8)	
Yes	90 (45.2)	
**Alcohol consumption in the previous 30 days**		
No	615 (41.9)	
Yes	852 (58.1)	
**Excessive alcohol use**		
No	1346 (91.7)	
Yes	121 (8.3)	
**Perceived health status**		
*12-Physical Component Summary (PCS-12)*		45.9 (±10.8)
*12-Mental Component Summary (MCS-12)*		44.9 (±11.6)
**PCP¹ accesses in the previous year**		
<12	782 (53.3)	
≥12	685 (46.7)	
**PCP¹ medical visits in the previous year**		
None	528 (36)	
<12	590 (40.2)	
≥12	349 (23.8)	
**Specialist visits in community health services in the previous year**		
None	890 (60.7)	
≥1	577 (39.3)	
**Private specialist visits in the previous year**		
None	778 (53)	
≥1	689 (47)	
**Emergency accesses in the previous year**		
None	1129 (77)	
≥1	338 (23)	
**Hospital admissions in the previous year**		
None	1279 (87.2)	
≥1	188 (12.8)	

¹ PCP = primary care physician

Regarding recommendations of PCPs on specifically reducing behavioral risk factors related to high blood pressure, 97% of the subjects claimed to have been advised on the use of drugs, almost 90% in the reduction of salt consumption, more than 80% in changing eating habits and augmenting physical activity, while fewer than three quarters were advised about reducing alcohol consumption (Table 2).

**Table 2:** Preventive health care delivered and actions to control high blood pressure among Italian adults

Preventive Indicators	Eligible		
	Subjects	**N**˚^†^	Percent*
**Screening and counseling on health habits**			
Having received screening for drinking problems	all subjects	1467	16
Having been asked about drug abuse history	all subjects ^§^	1467	6.6
Having received smoking cessation counseling visit within 3 months	all smokers identified as attempting to quit	68	19.1
Having received smoking cessation pharmacoterapy	all smokers attempting to quit who smoke more than 10 cigarettes a day	68	11.8
Having been asked about sexual activity history	all subjects ^#^	1467	9.7
**Physical examination**			
Having received blood pressure control at least once each year	all non hypertensive subjects	972	64.4
Having received evaluation of hearing difficulties at least every 2 years	subjects aged 65 and over	428	31.3
**Cancer screening services**			
Having received cervical cancer screening through PAP smear within the past 3 years	all sexually active women having an intact uterus	733	52.4
Having received PAP smear or colposcopy in low grade lesion within 6 months of the initial PAP smear	women having a PAP smear that shows a low grade lesion	2	50
Having received breast cancer screening through mammography in the past 2 years	women aged 50 to 70	297	51.5
Having been offered colorectal cancer screening tests (fecal occult blood test once each year or double contrast barium enema every 5 years or sigmoidoscopy or colonoscopy every 10 years)	subjects aged 50 to 80	740	21.3
Having received colorectal cancer screening with sigmoidoscopy or colonoscopy within in the past 10 years	subjects aged 50 to 80	740	14.2
**Adult immunization**			
Having been offered influenza vaccination annually	subjects aged 65 and over	428	90.2
	patients with specific conditions at risk	166	64.5
Having ever been offered pneumococcal vaccination	subjects aged 65 and over	428	26.2
	patients with specific conditions at risk	166	19.3

**The advice of doctor or other health professional to help lower or control high blood pressure**			

Having received advice to Change eating habits	hypertensive subjects	495	84
Having received advice to Reduce salt use	hypertensive subjects	495	89.5
Having received advice to Increase physical activity	hypertensive subjects	495	83.4
Having received advice to Reduce alcohol use	hypertensive subjects	495	73.9
Having received advice to Medication	hypertensive subjects	495	97

^† ^Subjects in the study who were eligible for the indicator; * Percent of eligible subjects who have received the recommended preventive services; ^*§ *^1079 subjects have answered to the question; ^# ^1073 subjects have answered to the question

Tables 2 and 3 show, respectively, the rates of delivery and the relationship between patients' characteristics and access to selected clinical preventive services stratified by multivariate analysis. Providing health habits preventive services was extremely low (less than 20%); those participants who were screened for drinking problems (16%) were significantly more likely to be males, excessive drinkers, less educated and frequent users of health services (PCP^' ^and public specialist' medical visits). Blood pressure check in nonhypertensives showed higher provision of preventive services compared to hearing difficulties in the elderly (64.3% versus 31.3%), and it was significantly more likely to be delivered to older subjects, the less educated and frequent users of health services. Rates of cancer screening were low for all types of cancer--approximately 50% of those eligible received breast or cervical cancer screening--but they were extremely low for colorectal cancer screening (21%). Even less likely to receive colorectal cancer screening were women, those with higher educational levels, current smokers, and those admitted to hospital; women, those more educated, married, workers, those with chronic disease and frequent users of private specialists were significantly more likely to receive cervical cancer screening, whereas breast cancer screening was significantly more likely delivered to younger women, those having attended PCP and or having been admitted to hospital admissions in the previous year. The recommendation for influenza vaccination was considerably higher compared with pneumococcal (respectively 90.2% versus 26.2% in the elderly, and 64.5% versus 19.3% in subjects with conditions at risk for severe complications); older patients with worse perceived physical health status were significantly more likely to benefit from both vaccines.

**Table 3:** Logistic regression models results

	Model 1	Model 2	Model 3	Model 4	Model 5	Model 6
	Blood pressure check	Colorectal cancer screening	Cervical cancer screening	Breast cancer screening	Influenza and pneumococcal vaccination	Screening for drinking problems

	Log-likelihood= -538.66, χ^2 ^= 188.34, p < 0.0001	Log-likelihood= -273.94, χ^2 ^= 56.52, p < 0.0001	Log-likelihood= -453.03, χ^2 ^= 108.43, p < 0.0001	Log-likelihood= -188.48, χ^2 ^= 34.49, p = 0.0001	Log-likelihood= -237.14, χ^2 ^= 17.75, p = 0.0131	Log-likelihood= -595.20, χ^2 ^= 100.52, p < 0.0001

	OR	OR	OR	OR	OR	OR
	(95%CI)	(95%CI)	(95%CI)	(95%CI)	(95%CI)	(95%CI)
Gender	-		-	-	-	
Male*		1.00				1.00
Female		0.44				0.42
		(0.27-0.71)				(0.31-0.57)
Age, continuous	1.03	1.02	-	0.95	1.06	-
	(1.02-1.05)	(0.99-1.05)		(0.91-1.00)	(1.01-1.10)	
Marital Status						
Married*	1.00	1.00	1.00	1.00		1.00
Other	0.74	0.73	0.33	0.60	-	0.72
	(0.53- 1.02)	(0.40-1.35)	(0.23-0.47)	(0.33-1.09)		(0.52-1.01)
Number in households, ordinal°	-	-	1.24	-	1.37	-
			(0.94-1.62)		(0.98-1.92)	
Education level, ordinal^#^	0.82	0.72	1.33	1.25	1.11	0.75
	(0.68-0.99)	(0.56-0.91)	(1.09-1.62)	(0.95-1.63)	(0.88-1.40)	(0.64-0.87)
Working activity	-	-		-	-	-
Housewife/student/unemployed/retired*			1.00			
Other			1.52			
			(1.05-2.21)			
Chronic disease		-		-	-	-
None*	1.00		1.00			
≥1	1.29		1.81			
	(0.91-1.84)		(1.23-2.67)			
PCS-12, continuous	1.02	1.02	1.01	-	0.98	-
	(1.00-1.04)	(1.00-1.04)	(1.00-1.03)		(0.95-1.00)	
MCS-12, continuous	-	1.01	1.01	1.02	1.01	-
		(0.99-1.03)	(0.99-1.02)	(1.00-1.04)	(0.99-1.03)	
Current tobacco use, ordinal^~^	-	0.69	1.20	1.34	1.24	-
		(0.49-0.98)	(0.98-1.47)	(0.93-1.92)	(0.84-1.84)	
Excessive alcohol use		-	-			
No*	1.00			1.00	1.00	1.00
Yes	0.75			0.25	0.50	2.19
	(0.47-1.22)			(0.06-1.04)	(0.14-1.80)	(1.37-3.51)
PCP^§ ^accesses in the previous year			-		-	
<1 time per month*	1.00	1.00		1.00		1.00
≥1 time per month	1.60	0.72		1.81		1.17
	(1.08-2.36)	(0.42-1.23)		(1.07-3.06)		(0.82-1.68)
PCP^§ ^medical visits in the previous year, ordinal^†^	1.74	1.32	1.11	-	-	1.30
	(1.34-2.25)	(0.95-1.82)	(0.88-1.40)			(1.04-1.62)
Specialist visits in community health services			-	-	-	
None*	1.00	1.00				1.00
≥1	1.49	1.34				1.46
	(1.07-2.08)	(0.84-2.13)				(1.08-1.98)
Private specialist visits,	-	-		-	-	-
None*			1.00			
≥1			1.37			
			(1.00-1.89)			
Emergency accesses in the previous year,	-	-			-	
None*			1.00	1.00		1.00
≥1			0.76	0.74		1.37
			(0.52-1.11)	(0.39-1.38)		(0.99-1.90)
Hospital admissions in the previous year			-		-	-
None*	1.00	1.00		1.00		
≥1	1.33	2.80		2.71		
	(0.79- 2.23)	(1.66-4.72)		(1.14-6.46)		

* reference category; ^§ ^PCP = primary care physician; ° Number in households: (none = 0, 1 = 1, >1 = 2); ^# ^Education level: (no formal education = 1, primary school = 2, secondary school = 3, high school or higher = 4);

^~ ^Current tobacco use: (not at all = 0, some days = 1, every day = 2); ^† ^PCP medical visits in the previous year: (none = 0, <1 time per month = 1, ≥1 time per month = 2)

## Discussion

In this study, among a representative sample of PCP' patients residing in Southern Italy, we had two original intentions: to examine the extent to which the healthcare system succeeds in providing clinical preventive services routinely recommended to adults [24-26] and to explore the disparities in their provision with relation to the patients' characteristics.

This study clearly demonstrates that the quality of preventive care in the target population falls far short of expectations; indeed, access to preventive health services is unsatisfactory even for interventions whose effectiveness is supported by broadly accepted scientific evidence, such as the commonly recommended cancer screening [27-35]. Consistent with other studies conducted in Italy [7-9,36], we found that cancer screening for early detection of breast and cervical cancers reached about half of target population but only one-fifth for colorectal cancer; these rates are discernibly much lower than those results reported in several studies conducted, particularly in the United States, that have shown variable results ranging from 58% to 89% for breast cancer, from 69% to 89% for cervical cancer, and from 31% to 57% for colorectal cancer [3-5,37-42]. Our results however were similar to those reported in studies conducted in vulnerable populations, such as the poor or uninsured or unenrolled in both Medicare and Medicaid programs [43-45], minority patients [46,47] and subjects who lack a regular care provider [48]. Coverage for cancer screening is probably so low due to the lack of an organised screening program in our Southern region; this is confirmed by studies conducted in Italy that have demonstrated an unmistakable imbalance in the provision of breast, cervical and colorectal cancer screening in Southern Italy [7-9].

We found only two clinical preventive services, blood pressure screening and the recommendation of influenza vaccination in subjects aged 65 and over, that showed satisfactory adhesion, reaching rates similar to or higher than most reported studies [3,36,49,50]. The high rate for screening of hypertension may correlate with many factors, such as simplicity and availability of blood pressure measurement, commonly performed with a sphygmomanometer, and to the global awareness of the importance of this clinical service not only by the individual physician but also by patients who did state in the interviews, that they often requested a blood pressure measurement.

The high rates of recommendation of influenza vaccination may reflect a positive attitude of PCPs, and are in accordance with findings of a previous study conducted by some of us in the same area, that indicated positive knowledge, attitudes and behaviours on vaccinations in the elderly in a large majority of PCPs [51].

Moreover, with respect to provision for screening and counseling on health habits services, we found that the rates were drastically lower than those reported in several studies conducted in the United States [3,15,16,52-54]. Several factors relating to the individual physician or patient may be mentioned as impediments to the provision of these services: lack of opportunity, lack of time due to other health concerns, the physician's negative attitude towards prevention or anticipated patient refusal; in defense, however, patients may not remember being asked about these conditions since they seemed not overly concerned about them.

Consistent with previous studies [43-47,55-59], we found significant disparities in the delivery of clinical preventive services. Primary care access measures, as well as proxies for the propensity to seek or request care, were all directly related to delivery of clinical preventive services, thus proving that more and better access to primary health care increases the likelihood of delivery of clinical preventive services. Therefore, our results strongly confirm the crucial role of PCPs in promoting preventive services, and agrees with previous studies conducted by some of us in the same area [60-62].

Regarding subjects' socio-demographic characteristics, in accord with several studies [3,47,55-59], we found that gender and age were related to disparities of delivery of certain clinical preventive services. Elderly men were more likely to have blood pressure checks and recommended routine influenza and pneumococcal vaccinations, but elderly women were less likely to receive mammography than younger women; moreover, females were less likely to have access to screening for drinking problems and to sigmoidoscopy or colonoscopy. The reasons for the observed demographic disparities are complex and vary according to outcomes. For instance, age-difference may be attributable to the awareness that the elderly are more susceptible to flu or pneumococcal disease [51] and hypertension [25], whereas early diagnosis of breast cancer has an attributable best benefit for younger groups [29]. Gender-related differences for screening for drinking problems may relate to perceived differences in alcohol intake between males and females [59] while colon cancer screening may reflect the preference among women to avoid invasive tests [58].

Contrary to expectations, level of education unevenly influenced access to clinical preventive services. A key finding was that women with higher level of education were more likely to have access to Pap smear but less access to colonoscopy and blood pressure check. A previous survey by the National Institute of Statistics (ISTAT) [63], showed that in Italy, particularly in the South, the use of the Pap test is more frequent among women with higher education level. Previous studies also show that disparities in levels of education (and its cognate, health literacy) is directly proportional to disparities in preventive care services [3,47,64-66]. We found, contrary to that, that higher level of education does not lead to a better access to preventive services as a whole, probably because high education level alone is not sufficient to ensure the necessary awareness of the importance of preventive care. In the healthcare system currently instituted in Southern Italy, the more educated the patient the less likely he is to consider the PCP as the central figure to consult when needed and this is confirmed by our results which show that more educated subjects prefer private specialists, and the PCP is consulted mainly for drug prescription[61].

The finding that screening for alcohol problem was significantly associated with alcohol intake is consistent with the results of a recent survey [67], that demonstrated that physicians try to motivate patients to address alcohol problems especially if they are heavy drinkers.

Finally, consistent with the national ISTAT data [68] revealing a distinctive North-South gradient, we found that the PCS-12 and MCS- 12 mean scores were lower than Italian general population norms, with a difference score of -2.7 and -5, respectively [22]; moreover Wallace et al [69] reports that the low scores are influenced by lack of social support and we may suggest that this sociological factor influences our current results as well. Of some interest, the health summary scores do not correspond significantly with the outcomes investigated, except for the PCS-12 score which influenced adult vaccinations. Holm et al [36] also found that poor health led most frequently to eventual vaccination.

Even tough we provide perceptive examinations of access and delivery of preventive care, our findings must be interpreted within the context of the study's limitations. The data were cross-sectional, patient-based and self-reported. Self reports, compared to other major data sources, such as medical records, may result in overestimates or underestimates of quality of preventive care. Stange et al [70], who compared medical records review and patient questionnaire methods to the gold standard of direct observation of the outpatient visit, showed that medical record review is preferable to patient questionnaire only with respect to the variables for which there are limitations in patient understanding or knowledge. Patient reporting is a more valid measure for other areas of service delivery, such as screening and counseling on health habits items, and reflect high sensitivity toward certain physical examination and cancer screening items that would be overt and memorable to a patient, such as Pap smear and breast and pelvic examinations; therefore, we do not think that our method of measuring the delivery of clinical preventive services may represent a substantial problem for the interpretation of our results.

The Rand Quality Indicators on health habits are recommended as a one time question and it may be that some patients would satisfy the quality indicator but may not remember that their PCP asked about them many years ago. In addition we do not have data on drinking problem or drug abuse and individuals may better remember services delivered for an actual problem rather than for prevention. Moreover, it should also be pointed out that if patients were only seen one time for an urgent issue it would be unlikely that they received these services.

Our results have significant implications for health care policymakers, since the indicators we used demonstrated to be a useful tool for monitoring access to clinical preventive services, assessing effectiveness of state-wide preventive health program, improve quality and reduce barriers to access to preventive health care through the implementation of future interventions in this population.

## Conclusion

For most Southern Italians, the healthcare system while universal and free is not doing a good job delivering vital clinical preventive services.

## List of abbreviations

PCP: primary care physician; SES: socioeconomic status; BRFSS: Behavioral Risk Factor Surveillance System.

## Competing interests

The authors declare that they have no competing interests.

## Authors' contributions

BM collected the data, and contributed to the data analysis and interpretation and wrote the first draft of the article. PR, AB and CGAN contributed to conception and design of the study, collected the data, and contributed to the data analysis and interpretation. MP designed the study, was responsible for the data analysis and interpretation, have been involved in revising the manuscript and have given final approval of the version to be published.

All authors read and approved the final manuscript.

## Pre-publication history

The pre-publication history for this paper can be accessed here:

http://www.biomedcentral.com/1471-2458/10/350/prepub

## Supplementary Material

Additional file 1 **Indicators of quality of preventive health care delivered to Italian Adults selected from RAND's Quality Assessment Tools**. Click here for file
