# Supplementary material for: The quality of preventive health care delivered to adults: results from a cross-sectional study in Southern Italy
Source: BMC Public Health. 2010 Jun 18;10:350. doi: 10.1186/1471-2458-10-350 (PMC2910674; doi:10.1186/1471-2458-10-350)
Supplement: Additional file 1 — Indicators of quality of preventive health care delivered to Italian Adults selected from RAND's Quality Assessment Tools. [file 1471-2458-10-350-S1.DOC]

**Indicators of quality of preventive health care delivered to Italian Adults selected from RAND's Quality Assessment Tools [2]**

| **Indicator text** | **Evidence*** |
| --- | --- |
| **Screening and counseling on health habits** |  |
| All patients should be screened for problem drinking. This assessment of pattern of alcohol use should include at least one of the following: use of a validated screening questionnaire (such as AUDIT, MAST or CAGE), quantity (e.g., drinks per day), binge drinking (e.g., more than 5 drinks in a day in the last month). | 3 |
| Patients should be asked about current or past use of intravenous drugs at least once. | 3 |
| All smokers identified as attempting to quit should be offered at least one additional smoking cessation counseling visit within 3 months. | 1 |
| All smokers attempting to quit who smoke more than 10 cigarettes a day should be offered pharmacotherapy except in the presence of serious medical precautions. | 1 |
| Patients should be asked if they have ever been sexually active. | 3 |
| Physical examination |  |
| Systolic and diastolic blood pressure should be measured on patients otherwise presenting for care at least once each year. | 3 |
| Patients over age 65 should be asked about hearing difficulties at least every 2 years. | 3 |
| Cancer screening services |  |
| Women who have not had a Pap smear within the past 3 years should have one performed (unless never sexually active with men or have had a hysterectomy for benign indications).^#^ | 2 |
| If a woman has a Pap smear that shows a low grade lesion (ASCUS or LGSIL), then one of the following should occur within 6 months of the initial Pap smear: repeat Pap smear or colposcopy. | 3 |
| Women aged 50 to 70 should have had a screening mammography performed at least every 2 years. | 1 |
| All average-risk adults age 50 to 80 should be offered at least one of the following colon cancer screening tests: FOBT (if not done in the past 2 years), sigmoidoscopy (if not done in the past 5 years), colonoscopy (if not done in the past 10 years), double contrast barium enema (if not done in the past 5 years). | 2 |
| Adult immunization |  |
| All patients aged 65 and over should have been offered influenza vaccine annually or have documentation that they received it elsewhere. | 1 |
| There should be documentation that all patients aged 65 and older presenting for care were offered pneumococcal vaccine at least once. | 1 |
| All patients under age 65 with any of the following conditions should have been offered influenza vaccination annually: living in a nursing home, chronic obstructive pulmonary disease, chronic cardiovascular disorders, renal failure, immunosuppression, diabetes mellitus, hemoglobinopathies (e.g., sickle cell). | 1 |
| There should be documentation that all patients in the following groups and otherwise presenting for care were offered pneumococcal vaccine at least once: chronic cardiac or pulmonary disease, diabetes mellitus, anatomic asplenia, and persons over age 50 who are institutionalized. | 1 |

* Level of evidence: 1=randomized trial; 2=nonrandomized controlled studies (e.g., case control); 3=observational studies/expert opinion.

^^ No upper age limit is recommended.
